# Coping Strategies and Burden Dimensions of Family Caregivers for People Diagnosed with Obsessive–Compulsive Disorder

**DOI:** 10.3390/healthcare10030451

**Published:** 2022-02-28

**Authors:** Marwa Abd El-fatah Ali El-slamon, Modi Al-Moteri, Virginia Plummer, Ahmed S. Alkarani, Mona Gamal Ahmed

**Affiliations:** 1 Nursing Department, College of Applied Medical Sciences, Taif University, P.O. Box 11099, Taif 21944, Saudi Arabia; marwa.a@tu.edu.sa (M.A.E.-f.A.E.-s.); m.motairy@gmail.com (M.A.-M.); 2 Department of Psychiatric and Mental Health Nursing, Nursing Faculty, Zagazig University, Zagazig 44519, Egypt; samasama200861@yahoo.com; 3 School of Health, Federation University, Berwick, VIC 3806, Australia; v.plummer@federation.edu.au

**Keywords:** burden, coping, family caregivers, obsessive–compulsive disorder, cross-sectional study

## Abstract

(1) Background: Obsessive–compulsive disorder (OCD) is a chronic mental disorder that can be a source of emotional, financial and/or social burden for family caregivers. Few studies have investigated family caregiving for patients diagnosed with OCD in relation to the coping strategies being used from a theoretical perspective. This study evaluated the burden and coping strategies of family caregivers for people diagnosed with OCD. (2) Methods: A cross-sectional study was conducted, in which 123 participants diagnosed with OCD and their caregivers were surveyed using three types of scales: obsessive–compulsive scale; coping scale; and burden scale. (3) Results: Of the participants with OCD and their caregivers, 53% and 31% were male and 47% and 69% were female, respectively. Around 80% of the OCD patients were considered young and their age ranged from 20–40 years old. Forty percent of caregivers in the current study reported a high burden level. The caregivers of those who had severe OCD symptoms had a lower coping level compared to the caregivers of those with less severe symptoms and those urban caregivers were able to better cope than rural caregivers. There was an association between OCD symptom severity and financial, work-related, social and family relationships, mental and health burdens for family caregivers. Meanwhile, the greater coping level of family caregivers, the lesser social and family, mental, and spouse relationship burden (*p* < 0.05); (4) Conclusions: The family caregivers of people diagnosed with OCD have specific aspects of burden and coping which require support by designing strategic interventions for family caregiver coping.

## 1. Introduction

Obsessive–compulsive disorder (OCD) is a common psychiatric disorder affecting 2–3% of the population worldwide: it is thus twice as prevalent as schizophrenia or bipolar disorder [1]. The disorder is characterized by obsessions as well as persistent, unwanted and disturbing thoughts that provoke distress and anxiety. In order to resist the persistent thoughts and reduce the level of anxiety, people with OCD tend to perform repetitive mental behaviors in an irrational way [2]. The effects of OCD may have repercussions in people’s daily activities, social and professional lives to the extent that these can cause a type of functional impairment. Stengler-Wenzke and colleagues [1] claimed that hospitalized patients diagnosed with OCD have greater functional impairment than hospitalized patients diagnosed with depression.

## 2. Theoretical Background

Due to the characteristics of OCD’s clinical presentation, family caregivers often need to change the routine of their own life to deal with the disorder of their family member—that is, compulsive symptoms. Such changes have placed great demands on family caregivers. Indeed, the literature has continuously reported the impact of caring for those diagnosed with OCD on caregivers’ well-being [3]. Although some families cope with providing care for relatives with mental disorders, others also find it hard. People exhibit numerous individual differences, and studies have shown a relationship between caregivers’ income, educational level [4], age [5] and their ability to manage their life when caring for people diagnosed with OCD. Some theories have been proposed to explain such variation. Of these theories is the “Folkman’s Transactional Stress and Coping Framework” developed by Lazarus and Folkman [6]. 

Folkman et al.’s [6] framework describes coping as an important approach that people sometimes use to cope with their physical or/and psychological distress that they perceive to exceed their personal resources [6]. The framework highlighted the presence of a mutual relationship between each burden, distress and coping strategy. However, according to the framework, perceived distress and personal resources vary among different people. In the current study, the Folkman et al. framework [6] will be used as a lens to view the burden, distress and the coping strategies of family caregivers of those people diagnosed with OCD and the possible variation. 

Family caregivers of people with OCD mainly reported a considerable amount of burden due the influence of the disease on their lives, and with little social and financial support, the burden might be intensified leading to feelings of extreme distress [5,6,7,8]. The burden of OCD on caregivers is well documented in the literature and was found to be greater than that of other mental disorders [7,8]. This probably due to the presence of OCD symptoms which leads to distress, anxiety and worry. Indeed, caregivers perceived the burden as greater if their family member had severe OCD symptoms and they had a more difficult time managing it [3]. The burden can be referred to as the difficulty of caring for someone as perceived by caregivers and the influence it has on their daily life [9,10,11]. A variety of physical, psychological, social and economic burdens could develop as a result of the caring role [7]. Feelings of distress, sadness and/or frustration and sometimes depression, were found to be critical indicators of severe burden [12] and could negatively influence a family caregivers’ health and functionality [7,11]. 

**Figure 1:**
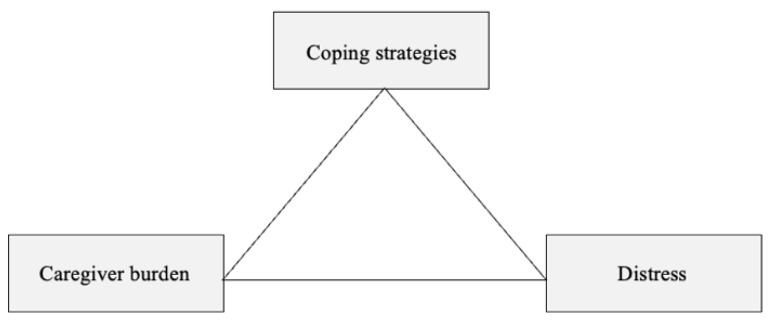
The Theoretical Framework of Caregiver Burden, Coping Strategies and Distress (adapted from Folkman et al. [6].

Under circumstances in which people encounter a particular source of stress, they first tend to conduct a logical analysis to identify whether a particular stimulus is relevant to their well-being [6]. This is followed by thinking about the alternative solutions and selecting the best plans [13]. This is referred to as coping strategies. Coping strategies are cognitive, emotional and behavioral efforts to overcome stress as well as reduce harm and pain [10]. This may involve the active management and/or alteration of an individual’s own environment. In the context of caring, caregivers generally assess the effect of the daily care of patients on their well-being, and the available support that could be used to deal with it. However, when a perception of distress resulting from care for patients with OCD exceeds the social, mental and financial supportive resources of caregivers, they tend to feel burden in multiple aspects of their daily lives [2]. This may include but is not limited to feeling overwhelmed, powerless, tired, experiencing health problems, physical, mental and financial exhaustion. It is therefore coping strategies that are key elements of distress management, which reduce the negative influence on family caregivers’ perception of burden (Folkman et al., 1986). The framework shown in Figure 1 presents the following sequence:

1. The burden of the caregivers of patients diagnosed with OCD is associated with coping strategies;
2. The burden of the caregivers of patients diagnosed with OCD is associated with distress (resulting from the worsening of the OCD symptoms);
3. Coping strategies have a mediating role in caregiver burden and distress.

Although there is an abundance of evidence available in the literature, there is still lack of a theoretical foundation underlying most of the evidence [14]. A study conducted by Suculluoglu-Dikici et al. [4] found that several factors affect burden. Suculluoglu-Dikici and colleagues recommended behavioral management to protect family health. In another recent study, caregivers who participated in an interview verbalized the different types of burdens they experienced and their need for educational training on effective coping strategies [7]. This implies the importance of having more evidence to contribute to the body of knowledge and build a general understanding of how caregivers manage their lives. This could be met by conducting studies that investigate the specific aspects of burden of the disorder on the caregivers and their possible correlates to caregivers’ sociodemographic factors. Identifying the most impacted aspects of a caregivers’ life, could help increase our understanding and therefore improve our planning for more specific interventions that lower the impact of caring for people diagnosed with OCD on family caregivers. Hence, this study aimed to evaluate the burden and coping strategies of family caregivers of those people diagnosed with OCD and a possible correlation.

## 3. Materials and Methods

A cross-sectional descriptive design was used. This study was conducted from July to August 2020. All participants were recruited from the outpatient units of two regional psychiatry hospitals located at Elsinbilawin city, in Dakahlia Governorate, Egypt. Both hospitals provide free and paid mental health services and both are specialized in the treatment of OCD among other mental disorders through inpatient and outpatient services.

### 3.1. Participants 

There were two groups of participants at both sites, family members and those diagnosed with OCD in the outpatient units of two regional psychiatry hospitals. Inclusion criteria were family caregivers and those diagnosed with OCD > 18 years of age and living together in the same place or at least living in the same geographic area as the person diagnosed with OCD; being available at the time of data collection; and family caregivers were the primary caregiver. The exclusion criterion was having OCD in addition to a comorbid psychiatric disorder. To obtain a significant result (*p* < 0.05) with enough power (80%), a minimum sample size of 84 was required to achieve a correlation coefficient of at least 0.3 [15]. A convenient sample technique was implemented. 

### 3.2. Data Collection Tool

Data were collected using four instruments including people with OCD and family caregivers’ demographic variables’ assessment chart. Demographic variables were collected included family members with OCD and the caregivers’ age, sex, residence, social status, education, work and socioeconomic level. 

The Yale–Brown Obsessive–Compulsive Scale was used to assess symptom severity for the disorder. The tool was designed and tested for reliability and validity by Goodman et al. [16] and consists of ten items. Each item was designed to ask the people with OCD about their symptoms. For each item, the people with OCD rated the degree to which the item applied during the previous week on a scale of 0–4. A total score from 8 to 15 indicated ‘Mild OCD’; 16–23 indicated ‘Moderate OCD’; 24–31 indicated ‘Severe OCD’; and 32–40 indicated ‘Extreme OCD’. 

Burden Scale [17] was used to assess the burden of chronic mental illness on the family caregivers. The tool consisted of 40 items. Each item was rated on a 3-point scale (not at all, to some extent, and very much). The higher the total score, the greater the burden. The tool includes six specific aspects of burden: financial aspect (4 items); work-related aspect (4 items); social and family relationship aspect (12 items); mental health aspect (9 items); physical health aspect (5 items); and spouse relationship aspect (6 items). The tool has been found to be statistically valid and reliable [17]. The instrument has a Cronbach’s alpha of 0.73.

The Coping Scale [18] assessed the caregivers’ use of both behavioral and cognitive-emotional strategies for dealing with stressful events. The strategies were guided by caregivers’ appraisals of the events and their beliefs in relation to their ability to effectively cope with the stressful event. The scale included 13 strategies, consisting of (7 items) reflecting cognitive appraisal; (4 items) representing behavioral methods of coping; and (2 items) managing. Each item was rated on a 4-point scale which ranged from ‘mostly true’ to ‘not at all true’. The scale validity and reliability were established by Hamby and colleagues [18].

### 3.3. Data Collection Procedure

Prior to conducting the study, the outpatient appointment records from the two regional psychiatry hospitals where people diagnosed with OCD were scheduled for a follow-up appointment with psychiatric practitioners were reviewed by the researchers. Of the 304 patients listed in the appointment records, 142 persons diagnosed with OCD had an appointment in the outpatient clinics from July to August 2020. The researchers contacted the caregivers of the 142 patients by telephone and invited them and their relative patients to participate in the study. Among the 142 pairs, 130 agreed to participate (130 caregivers and 130 patients with OCD). Researcher lost seven participants during the follow up. 

As soon as the participants reached the outpatient psychiatric clinics, they met one of the researchers who accompanied them to a vacant office, provided by the hospitals, to maintain privacy during data collection process. Soon after reaching the data collection place, the researchers explained the purpose of the study and the methodology. The participants were encouraged to ask questions and their concerns and queries were resolved prior to the commencement of data collection. Both patients and their caregivers were informed that they had the right to withdraw at any point of the study. They were also advised to inform the researchers if they experienced any mental or physical discomfort. Finally, informed written consent was obtained from both the person diagnosed with OCD and their family caregiver. 

The demographic variables were collected for both the participants with OCD and their family caregivers; the Yale–Brown Obsessive–Compulsive Scale was completed by participants with OCD; while the Burden Scale and Coping Scale were completed by the caregivers. The completed questionnaires were reviewed by the researchers. Of the 123 participants who agreed to take part in the current study, seven participants with OCD and with mental health comorbidities that could affect the coping and the burden of their family caregivers were excluded. Ten people with OCD and their family caregivers were excluded as the caregivers failed to complete the self-reported scales. Six family caregivers were excluded as they were not primary caregivers. The remaining 100 participants with OCD and 100 family caregivers who met the research criteria were enrolled in this study.

### 3.4. Ethical Approval

This study was approved by the Scientific Research Ethical Committee (SREC), Zagazig University, Egypt: research protocol SREC 72/1401. This study was conducted in accordance with the Declaration of Helsinki. 

### 3.5. Maintaining Confidentiality

The voluntary nature of the participation, anonymity and confidentiality of participant information was continuously maintained. No identifiable data were collected in the surveys. The collected anonymous surveys were stored according to the guidelines of the scientific research committee and could never be re-identified. The participant number and phone number used during the recruitment process were stored safely.

### 3.6. Data Analysis

The data were analyzed using SPSS 23.0 for Windows (SPSS Inc., Chicago, IL, USA). Descriptive statistics, chi-square test or Fisher’s exact test was used when appropriate. Spearman’s rank correlation coefficient was used to assess the relationships between study variables. 

## 4. Results

The demographic characteristics of the participants are summarized in Table 1. Around 50% (*n* = 49) of those diagnosed with OCD were aged between 30 and 40 years old, while 44% (*n* = 44) of the family caregivers were aged over than 40 years old. Sixty-nine percent of the studied caregivers were female and 46% (*n* = 46) were spouses. Approximately 70% (*n* = 71) of the caregivers were employed in a paid job and were of middle socioeconomic status. Among the caregivers and care recipients living in rural areas, 40 (40%) of the caregivers lived with the care recipient. Meanwhile, among the caregivers and care recipients living in urban areas, 57% (*n* = 57) of the caregivers did not live with the care recipient but lived in the same geographic area, while 3% lived with their care recipient. Approximately 50% of those with OCD (*n* = 52) and caregivers (*n* = 44) had high school education. 

### 4.1. Descriptive Statistics for Family Members with OCD Symptom Severity and for Caregivers’ Burden and Coping Score 

The OCD score of the studied participants ranged from 16 to 40 with a mean ± SD (29.8 ± 6.9); specifically, 43.0% of the studied participants with OCD reported experiencing highly severe OCD symptoms in the last week. The burden score of the studied caregivers ranged from 52 to 117 with mean ± SD (94.7 ± 11.9) and 40.0% of them reported a high burden level. Specifically, the social and psychological burden ranged from 18 to 36 with mean ± SD (26.5 ± 4.2) and 12–27 with mean ± SD (22 ± 2.8), respectively. Meanwhile, the coping score of the studied caregivers ranged from 13 to 42 with the mean ± SD (30.4 ± 8.2) and 76.0% of them reported moderate coping level. 

### 4.2. Relationship between Symptom Severity of Family Members with OCD and Their Caregivers’ Burden and Coping Score 

Pearson’s correlation coefficient was carried out to assess the relationship between those with OCD and the burden level of the caregiver *p* < 0.0001 and their coping level. There was a statistically significant relationship between the OCD level of family members with OCD and the burden level of the caregiver *p* < 0.0001 and their coping level *p* = 0.005. It seems that people who have a severe or highly severe level of OCD had caregivers who had a severe level of burden and low coping level. When the relationship between the burden and coping levels was further investigated, there were statistically significant relationships between the burden level of the caregiver and the coping level (*p* = 0.0001). Specifically, family caregivers who had a severe burden level, were found to have the lowest coping levels (Table 2).

### 4.3. Relationship between Caregivers’ Burden and Coping Score and Demographic Data 

Analysis of the relationship between the coping level of the studied caregivers and their demographic profiles revealed several significant findings. The relationship between coping strategies and the place of living, for those caregivers and care recipients who live in urban areas, revealed that coping was high among caregivers who live in the same geographic area as (*p* = 0.007), while the burden was significantly higher among caregivers who live with the care recipient in rural areas (*p* = 0.09). Furthermore, the relationship between the burden level of the studied caregivers and their socioeconomic status and educational level demonstrated a statistically significant relationship (*p* = 0.017 and *p* = 0.004, respectively). This indicates that caregivers of high socioeconomic status suffered from a moderate burden level, while those caregivers of low socioeconomic status suffered from a severe burden level.

### 4.4. Relationship between OCD Severity and Aspects of Burden 

Table 3 presents a summary of the relationship between OCD severity and aspects of burden (financial aspect, work-related aspect, social and family aspect, mental health aspect, physical health aspect and spouse relationship aspect). Greater OCD severity was associated with greater financial, work-related, social and family, mental and health burden. Meanwhile, a greater coping level of the caregivers indicated that there was less burden on their social and family lives, less mental burden and less burden on their spouse relationship (*p* < 0.05). 

### 4.5. Results under the Lens of the Theoretical Framework 

**Figure 2:**
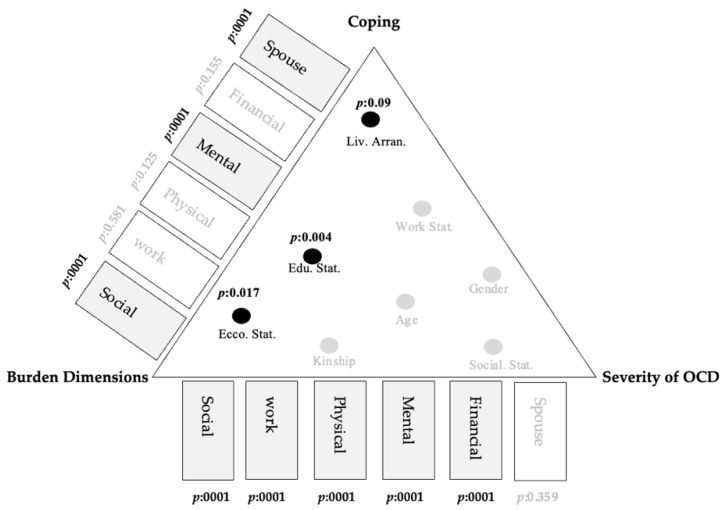
Results of the analysis—effects of OCD symptoms on caregiver burden and coping levels. Notes: Liv. Arran. = living arrangements; Social Stat. = social status; Edu. Lev. = education level; Work Stat. = work status; Eco. stat. = socioeconomic level; Kinship = kinship.

The caregiver burden, coping strategy and OCD symptom severity in relation to the demographic variables were entered into the model to investigate the mechanisms linking the caregiver burden to the coping strategy and the OCD symptom severity and possible correlation to demographic data. This study demonstrated the findings in the following sequence: first, this study demonstrated the relationships between the burden dimensions and coping strategies of the caregivers. Second, this study demonstrated the relationships between the burden dimension and OCD symptom severity. Third, the study demonstrated the relationships between demographic data and the burden and coping levels. The details shown in the model are presented in Figure 2.

## 5. Discussion

This study was an attempt to assess the burden aspects and the coping levels of family caregivers and investigate whether there was a possible correlation. Despite the fact that several studies have been conducted worldwide to evaluate the burden and the coping levels of caregivers of people diagnosed with OCD, the current study is one of the few to have investigated family caregiving for OCD people from a theoretical perspective to assess the specific aspects of burden for OCD’s caregivers. 

Burden is a multi-aspect construct [8]. There is a lack of studies showing the dimensions of caregiver burden, coping strategy, and distress resulting from the severity of OCD symptoms in Middle East countries, where family obligations are greatly important [19]. The findings of the current study not only support the Folkman et al. [6] framework, but also provide insight for policy makers to develop appropriate interventions to address caregivers’ needs. Furthermore, nurses’ interaction with patients, their families and other caregivers puts them in an important position to help caregivers reduce the negative impact of the disease on their family well-being [20].

This study shows that a greater severity of symptoms in people with OCD indicates a greater burden level on the caregiver. Such findings might be somehow justified from a cultural view. Arab cultures are generally based on an extended family system, and entire family members often live together in the same geographical area or sometimes in the same house. Traditionally, a healthy relative is mandated to take the responsibility for caring for those family members who are mentally unwell [19]. Caregivers’ burden from caring for mentally ill relatives has generally been significantly reported in the literature, and several studies showed the presence of variation in the overall level of burden observed worldwide [21,22].

It is not just mental diseases such as depression [23] and schizophrenia [24,25] that place excessive demands on family caregivers. The literature has shown that the caregivers for those suffering from other medical or physical diseases such as dementia [26], stroke [27] and physical disability [28] have also experienced a negative impact on their lives. Interestingly, giving care to a family member with mental, medical disorders and disabilities is not always seen as a burden [3]. Caregivers may also perceive some positive aspect of giving care in the form of becoming closer and more sensitive to the needs of their family member [3,29,30]. Part of this positive sentiment could be attributed to the adoption of successful coping strategies. Indeed, some studies have shown that caregivers have used coping strategies with encouraging results in terms of decreasing the burden level [13]. 

The current study revealed that there is a significant association between the caregivers’ burden level and their socioeconomic status. This result is consistent with the literature as the burden level tends to be high in individuals of lower education and socioeconomic status [31,32]. In this study, it appears that caregivers who live in urban zones in the same geographic area are more able to cope with their role of caring for their relative with OCD. An explanation may be that those from urban areas may have more knowledge about the disorder due to their higher education level, more access to support from health professionals and show a more positive attitude and better ability to cope with their negative feelings [30]. Furthermore, it has been well known that cultural differences and related factors can affect public perceptions and attitudes surrounding those with OCD, leading to different caregivers’ experiences [7].

In the current study, greater OCD severity was associated with greater financial burden. This may be attributed to the significant role of caregivers as a worker and supplier of financial requirements, leading to great economic burden in addition to other financial strains such as the costs of psychiatric medications and limited coverage of free treatment by the governmental policies. 

In this study, the severity of OCD has a negative impact on caregivers’ general physical health [31,33] and mental health [34]. Oza et al. [35] concluded that the burden of care has a strong positive correlation with distress and psychiatric morbidity in the caregiver of family members with OCD. This may imply a need for more focus concerning marital relationships and OCD.

Caregivers’ social and family relationships can be influenced by OCD symptoms if coping strategies are not sufficient. Caregivers may assume responsibility for many daily activities that people with OCD are unable to undertake. Caregivers’ abilities to maintain a social life and care for the remaining family members will decrease [36,37]. This could lead to an impaired social life, social supports and dysfunction in families. Koujalgi and colleagues [37,38] recommended appropriate assessment and psychosocial management for families with a member diagnosed with OCD. Specifically, Koujalgi and colleagues [37] stressed the importance of implementing educational programs, teaching family members problem-solving skills and seeking out social support to help the family to more effectively cope. 

## 6. Study Implications

The current study findings may have several implications for healthcare professionals and family caregivers. For instance, educational interventions and initiatives are likely to be implemented to support the family caregivers of patients diagnosed with OCD. Indeed, teaching family caregivers about the benefits of adopting coping strategies will enhance their spouse, social and family relationships as well as their mental health. Psychological advisors could help family caregivers obtain the appropriate coping strategies to address their spouse, social, family and mental burdens. Furthermore, the benefits of coping strategies may extend to improving financial and social services and support to allow family caregivers to cover the financial burdens of providing care for patients. Healthcare policy makers may promote training and educational programs in the community as well as healthcare settings that aim to improve the well-being of family caregivers, particularly in regional areas where isolation may contribute to breakdowns in the caregiver–family member relationship.

## 7. Study Limitations

This study had several limitations. First, randomization was not maintained during data collection, and this can limit the generalizability of the study findings. This study’s researchers admit the potential bias of using the self-report method [39]. Furthermore, due to the use of cross-sectional designs, the relationships between burden, coping strategies, and OCD symptoms cannot be definitely determined. Although this issue has been managed by examining the variables through the lens of Folkman’s Transactional Stress and Coping Framework” [6], longitudinal studies should be implemented in future studies. Since the burden and coping level are variables that are time sensitive and could change during specific circumstances, collecting the data shortly after the end of the first wave of the coronavirus pandemic may have influenced the results of the current study [40]. 

## 8. Conclusions

Around 40.0% of caregivers in the current study reported a high burden level. The burden of caring on the behalf of family caregivers for people diagnosed with OCD is the most critical factor that affects almost all aspects of life for caregivers—financial, work-related, social and family, mental health and physical health. The study findings highlight the role of coping strategies on some burden dimensions. Specifically, illiterate relatives caring for patients with OCD (*p* = 0.004) who are suffering from some financial constraints (*p* = 0.017) and live in rural areas (*p* = 0.09) were more likely to have an increase in financial, social, mental physical and work-related burden. Family caregiver burden should be minimized via assessment and psychosocial supportive programs. Due to the important position of the nurses interacting with patients, their families and other caregivers, they have an important role in teaching patients and their relatives some simple but important techniques on how to manage the negative impact of the disease on their well-being. Moreover, family caregiver needs should be an important part of the nursing care plan. Future studies are needed to investigate the impact of different clinical manifestations of OCD on caregivers’ burden and coping.

## Figures and Tables

**Table 1:** Demographic characteristics of participants with OCD and caregivers.

	*Parameters*	Participants with OCD *n (%)*	*Caregivers* *n (%)*
Age (years)	20-	30 (30.0%)	19 (19.0%)
	30–40	49 (49.0%)	37(37.0%)
	>40	21 (21.0%)	44 (44.0%)
Sex	Male	53 (53.0%)	31(31.0%)
	Female	47 (47.0%)	69 (69.0%)
Living arrangements	Rural: live with the care recipient	40 (40.0%)	
	Rural: do not live with the care recipient but live in the same geographic area	0.00	
	Urban: live with the *care recipient*	3 (3.0%)	
	Urban: do not live with the *care recipient* but live in the same geographic area	57 (57.0%)	
Social status	Single	27 (27.0%)	8 (8.0%)
	Married	54 (54.0%)	83 (83.0%)
	Divorced	13 (13.0%)	3 (3.0%)
	Widow	6 (6.0%)	6 (6.0%)
Education level	Illiterate	8 (8.0%)	8 (8.0%)
	Elementary	10 (10.0%)	6 (6.0%)
	High school	52 (52.0%)	44 (44.0%)
	University	30 (30.0%)	42 (42.0%)
Work status	Working	39 (39.0%)	70 (70.0%)
	Not working	61(61.0%)	30 (30.0%)
Socioeconomic level	Low	23 (23.0%)	16 (16.0%)
	Middle	65 (65.0%)	71 (71.0%)
	High	12 (12.0%)	13 (13.0%)
Kinship	Mothers		26 (26.0%)
	Brother/sister		25 (25.0%)
	Spouse		46 (46.0%)
	Fathers		3 (3.0%)

**Table 2:** The relation between the OCD symptom severity of participants with OCD, caregivers’ burden and coping scores.

Variables	Obsessive–Compulsive Level of Studied Participants						χ^2^	*p*
	Moderate *n* = 24		Severe *n* = 33		Extremely Severe *n* = 43			
	No.	%	No.	%	No.	%		
Burden level of caregivers								
Low burden	4	16.7	0	0.0	0	0.0		
Moderate burden	18	75.0	16	48.5	22	51.2	22.6	0.0001(S)
Severe burden	2	8.3	17	51.5	21	48.8		
Coping level of caregivers								
Moderate	20	83.3	30	90.9	26	60.5	10.4	0.005(S)
Low coping	4	16.7	3	9.1	17	39.5		

**Table 3:** Correlation between the OCD symptom severity of OCD participants, caregivers’ burden dimensions and coping scores.

Burden Dimensions	Obsessive–Compulsive Score of Participants		Coping Score of Participants Caregiver	
	(r)	*p*	(r)	*p*
Financial aspect (4)	0.348 **	0.0001	−0.143	0.155
Work-related aspect (4)	0.380 **	0.0001	−0.056	0.581
Social and family relationship aspect (12)	0.347 **	0.0001	−0.495 **	0.0001
Mental health aspect (9)	0.497 **	0.0001	−0.360 **	0.0001
Physical health aspect (5)	0.344 **	0.0001	−0.155	0.125
Spouse relationship aspect (6)	0.093	0.359	−0.399 **	0.0001

** (r) correlation coefficient significant *p* < 0.05.

## Data Availability

The data presented are included in this study; additional data may be provided by the corresponding author on request.
